# A New Architecture for Customizable Exergames: User Evaluation for Different Neuromuscular Disorders

**DOI:** 10.3390/healthcare10102115

**Published:** 2022-10-21

**Authors:** Martina Eckert, Alicia Aglio, María-Luisa Martín-Ruiz, Víctor Osma-Ruiz

**Affiliations:** 1 Group on Acoustics and MultiMedia Applications (GAMMA), Centro de Investigación en Tecnologías Software y Sistemas Multimedia Para la Sostenibilidad (CITSEM), Universidad Politécnica de Madrid (UPM), 28031 Madrid, Spain; 2 InnoTep Research Group, ETSIS de Telecomunicación, Universidad Politécnica de Madrid (UPM), 28031 Madrid, Spain

**Keywords:** exergames, Kinect, neuromuscular disease, physical disability, rehabilitation, serious games, virtual reality rehabilitation (VRR)

## Abstract

This paper presents a modular approach to generic exergame design that combines custom physical exercises in a meaningful and motivating story. This aims to provide a tool that can be individually tailored and adapted to people with different needs, making it applicable to different diseases and states of disease. The game is based on motion capturing and integrates four example exercises that can be configured via our therapeutic web platform “Blexer-med”. To prove the feasibility for a wide range of different users, evaluation tests were performed on 14 patients with various types and degrees of neuromuscular disorders, classified into three groups based on strength and autonomy. The users were free to choose their schedule and frequency. The game scores and three surveys (before, during, and after the intervention) showed similar experiences for all groups, with the most vulnerable having the most fun and satisfaction. The players were motivated by the story and by achieving high scores. The average usage time was 2.5 times per week, 20 min per session. The pure exercise time was about half of the game time. The concept has proven feasible and forms a reasonable basis for further developments. The full 3D exercise needs further fine-tuning to enhance the fun and motivation.

## 1. Introduction

People with physical disabilities caused by injury or illness need to exercise to regain or maintain their fitness level, in order to prevent muscle loss. Due to their limited possibilities to access regular sports activities, they have to undergo personalized physiotherapy, which is often a costly and time-consuming burden [1]. In addition, it often requires the patient to perform additional exercises at home that cannot be supervised by a professional [2]. Virtual reality rehabilitation (VRR) is a promising solution: serious games (SG) based on 3D motion capture provide the possibility of additional physical training in combination with conventional treatments. Furthermore, they motivate, which improves performance, and an individual’s progress can be monitored [3]. However, more efforts are needed in this field to make these games really attractive and ensure they are adapted to the specific needs and possibilities of the patient [4].

Many studies on VRR can be found in the literature, but most are made for older people with common diseases like a stroke or Parkinson’s disease. Some examples are the navigation system [5] developed by Pool et al., “ReHabgame” [1], a VRR system with three different scenarios, three low-cost games presented by Seo et al. [6], the therapeutic game system “Rehab @ Home” [7] for fine and coarse arms and shoulder movements, or “Motion Rehab AVE 3D” [8] for the motor and balance rehabilitation of patients who have suffered mild strokes.

However, there are very few applications for children and young adults, who are more likely to play video games. Most of the existing studies have been carried out for cerebral palsy (CP) patients. They form, along with patients with neuromuscular diseases (NMD), the main group of young people with motor dysfunction [3,5,9,10,11,12]. However, NMD patients, e.g., those suffering from Duchenne muscular dystrophy (DMD) or spinal muscular atrophy (SMA), often cannot use the same applications as CP patients. DMD patients progressively lose their muscle strength and are unable to perform frequent and rapid movements. The literature recommends for DMD aerobic exercises without exceeding 20% of the maximum voluntary contraction and should gradually increase the intensity [13,14]. On the other hand, for SMA patients, whose disease is caused by a disturbed muscle control through the nerves, strengthening is not harmful to the muscles and high repetitions are beneficial [15]. Therefore, each NMD patient has different demands and parameters, like the frequency or strengthening factors of movements, which are usually not adjustable in commercial games.

In addition to lacking the adaptability to different types of disorders and features to personalize therapies, VRR still does not pay enough attention to the possibilities offered by a good game design; while commercial exergames are long, engaging, and completely immersive, rehabilitative exergames consist mostly of mini-games that are mere translations of conventional exercises into a virtual environment. As far as we know, there are practically no examples of “full-play exergames”, as we call games that are based on a story, have a protagonist, and use elements and mechanisms that take the patient into the world of the game.

Some examples of mini-games are: “JeWheels”, a wheelchair game for collecting coins with both arms [16], a game for collecting fruits for posture control training [17], and “IGER” (intelligent game engine for rehabilitation) [18], a system that offers a series of mini-games for posture and balance rehabilitation, monitored remotely by a therapist.

The commercially available VRR solutions are also based on individual mini-games, e.g., the previously mentioned software “MIRA” from MiraRehab [19] or “VirtualRehab” from Evolv [20], which contains various assessment modules, guided exercises and different types of exergames for a variety of neurological diseases. Other examples are “REHABILITY” [21], which allows rehabilitation exercises to be carried out remotely and autonomously under medical supervision, and “Mitii” (move it to improve it) [22], which has been analyzed in several studies, e.g., in [11,12,22].

However, researchers are aware and agree that simplicity and the lack of cognitive challenges leads to boredom and that cognitive tasks have a positive effect on motivation, and therefore on one’s physical performance and progress [20]. Various authors, such as Mihelj et al. [23], Zimmerli et al. [24], or Gorsic [25,26], showed this effect. Mihelj et al. stated that “a scenario targeting cognitive and motor training would have to be adaptable to specific patient capabilities defined by cognitive and motor deficits. This would make the adaptation of task difficulty more complex but could lead to better improvements in motor and cognitive capabilities”.

Following these ideas and in order to close the identified gaps, we developed the first prototype of a fully functional rehab exergame “Phiby’s Adventures” [27] as part of our Blexer project (Blender Exergames). It is the sequel to four individual mini-games (four scenes of upper limb exercise) that have been tested for their ease of use in [28] and are now included in a full game with a main character and a meaningful story. The game architecture combines the exercises in a modular way. They can be arranged in different sequences and frequencies or replaced by any others.

Our hypothesis is that the exercises, when integrated into a larger VRR game, which is generic but customizable, will similarly attract and engage people with very different physical conditions if the game is well adjusted to their abilities. In this case, we assume that people would focus mainly on the game and forget about the physical effort. For this reason, we tested our prototype on a population of 14 NMD patients who are affected differently. They ranged from the mildly affected, who can play standing, to wheelchair players, who only have a limited arm movement.

The population was similar to our previous tests in [28], but the results cannot be compared directly because (a) the exercises, although based on the same movements, have been significantly refined to ensure a better functioning, (b) the difficulty is adjusted through “Blexer-med”, and (c) the users play at home and decide the time and duration of their play.

This study is not a clinical trial and no health-related criteria are evaluated. The authors want to propose new ways of rehabilitation by presenting the next step beyond mini-games. They do not want to prove that complex games are better, but rather show new ways in which existing mini-games can be integrated into a larger environment in order to increase user interaction. The exercises tested here are only examples and can be replaced by any others. 

The paper is structured as follows: in Section 2.1, we briefly explain the “Blexer-med” system environment and present the exergame used for the intervention and study (a complete description of both can be found in [29]). Section 2.2 describes the methodology used for the recruitment, evaluation, and analysis. The results are shown in Section 3 and discussed in Section 4.

## 2. Materials and Methods

### 2.1. System Environment

**Figure 1:**
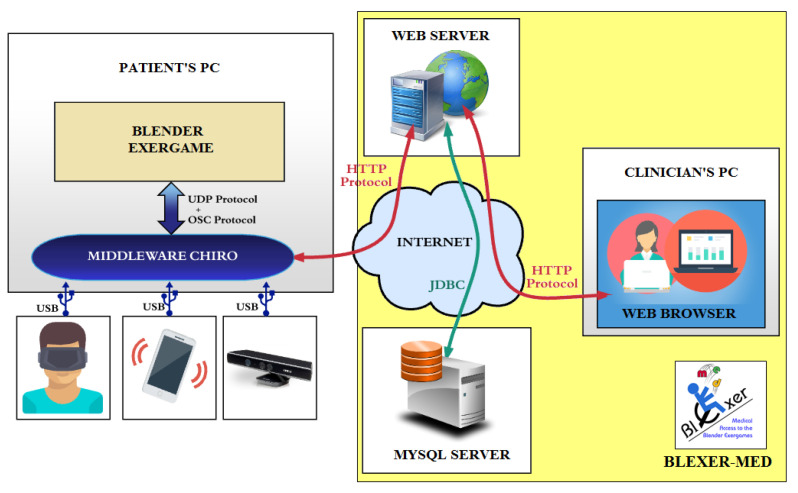
“Blexer-med” system environment. Left: patient’s side at home; right: clinical web and database.

The system environment “Blexer-med”, which is used for the intervention presented in this document, is shown in Figure 1 and has been described in detail in various previous publications [27,28,29,30]. 

It consists of two parts: the user side (users with Kinect X360 and personal computer for middleware and game(s)) and the clinical side (the database and webserver for therapists). The middleware “Chiro” [30] establishes the connection between the games with the web platform and the camera. Clinicians can configure the game settings and monitor users’ results over the web.

**Figure 2:**
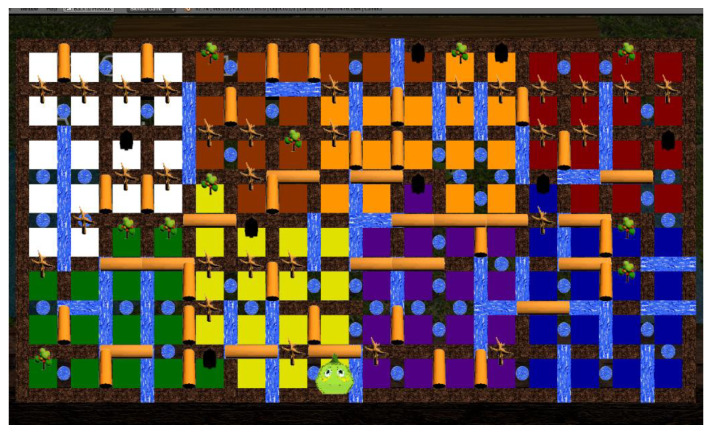
Map of the game environment. In each cell, the path can be chosen through one of the four obstacles: tree, lake, river, or trunk.

The web can host multiple games. Our first fully functional prototype, “Phiby’s Adventures v1”, is a third-person semi-3D video game based on a 2D map of 16 × 8 cells (see Figure 2), which represents a valley.

Gameplay: the main character “Phiby” starts in one cell and must find a way out of the valley without knowing the surroundings. After passing through each cell, the user can visualize the already explored part by lifting one hand. The cells are surrounded by four types of obstacles (a tree, river, lake, or trunk) that Phiby must overcome: climb a tree, cross a river by boat, dive through a lake and eat plankton, or chop down a few tree trunks. 

**Figure 3:**
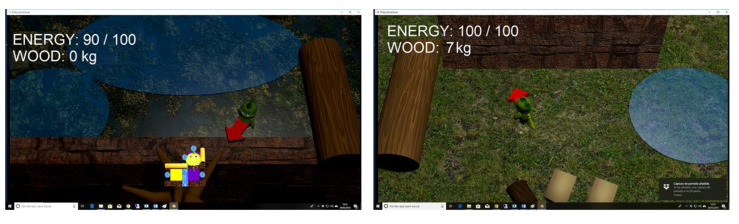
Screenshots of two cells on the map. Left: a tree trunk, a wall (no passage), and a lake. The energy is 100% and Phiby gained 7 kg of wood in the previous exercise. Right: Phiby can choose between two different lakes or chopping wood. The energy is 90% and the map shows the explored environment.

The route can be freely chosen, even back to a previous cell. Inside a cell, Phiby is controlled by horizontal hand movements. When he reaches an obstacle, a new scene opens that contains the exercise that must be performed to pass the obstacle (row a boat, climb a tree, etc.). Figure 3 shows two cells of the game, the right one containing the map of the already explored area.

**Figure 4:**
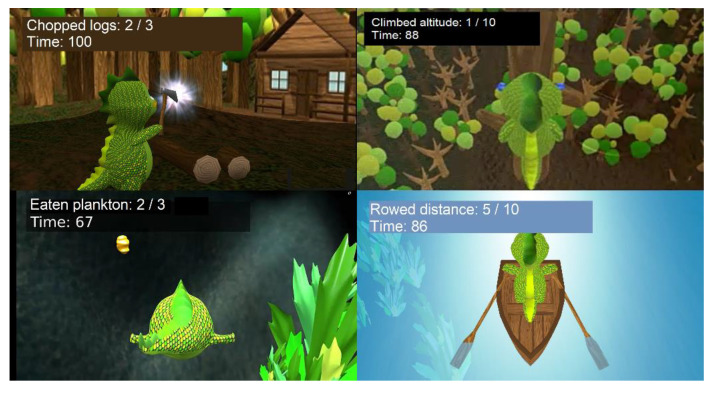
Exercises from left to right, up–down: “Chop the wood”, “Climb the tree”, “Dive and eat”, and “Row the boat”.

Exercises: the curse of the game contains four exercises, as shown in Figure 4: diving, chopping wood, rowing, and climbing a tree. Rowing and climbing are based on simple movements with both arms: a simultaneous forward and backward movement and an alternating up and down movement. Chopping is a more complex up–down movement with one arm (only the right one was available at the time of the experiments): first, the arm has to be raised and held at the highest point for a few seconds in order to “charge” the axe. The state of the charge is represented by a growing circle and a flash of light indicates when the axe is ready (Figure 4). Now the arm can be lowered. If the player drops the arm too soon, the trunks will not break.

In the diving exercise, the user trains the upper body through forward, backward, and sideways movements. Those are needed to control Phiby to reach the planktons. Compared to the others, this exercise is a real 3D experience as the player can move freely in the lake and search for plankton.

Game mechanics: the exercises use up Phiby’s energy, but he can recover by finding apples or apple trees. With the wood obtained in each chopping exercise, bridges or huts can be built. By crossing a bridge, Phiby can return across a river without rowing. A hut is a place where the state of the game is saved so that the player can continue at this point in the next session. There are eight cells in which huts can be built. As long as no hut has been built, the player must start over in each session.

With these types of game elements, users are able to think, plan, and decide independently, which adds a lot of variety to the game. The cell structure was chosen to achieve the following: (a) an integration of the four exercises tested in [28] into a meaningful story; (b) enable the user to freely choose between different exercises; and (c) a software that runs without problems on standard personal computers. More sophisticated gaming environments often require special graphic processors and a large memory capacity.

The authors do not regard the exercises as mini-games in the usual sense. Mini-games are individual short games that are often found in larger games, but generally have completely different game elements and have no relation to the main game (e.g., puzzles that are offered to score additional points). In our case, the exercises are a necessary part of the game and must be done in order to achieve the final goal. If players fail an exercise, i.e., the available time is running out before reaching the target, they remain in the same cell and can try again or choose a different route. In this way, the user decides how often he/she does the same exercise. It mostly depends on luck how quickly someone can find the way to the end of the game. The frequency of each exercise is therefore completely different for each player. Furthermore, the architecture allows one to interchange the exercises by others if they are more useful or rehabilitative for the user.

Therapeutic adjustments: the game can be played sitting or standing, depending on the participants’ capacity. The clinician uses the website to determine the difficulty of the exercises by adjusting the width of the rivers, the height of the trees, the number of trunks to be chopped, and the amount of plankton to be eaten. A time limit can be set in seconds for all exercises so that the level of difficulty increases with more movements or less time. The three arm-exercises (rowing, climbing, and chopping) involve amplification based on an initial calibration that measures the range of motion. When a user ends a session, the middleware automatically transfers the achieved scores and times to the database. The therapist can then observe the performance on the web interface and, if necessary, adjust the difficulty for the next session.

### 2.2. Evaluative Tests

The quantitative focus group design was selected to extract the motivations and barriers to the exergame system described before. Informed consent was obtained from the participants and their parents. Approval for the study was obtained from the Ethical Committee of the Universidad Politécnica de Madrid.

#### 2.2.1. Participants

We focused our study on children and adolescents with neuromuscular disorders. They were recruited in Madrid (Spain) by distributing leaflets and contacting various foundations and associations, hospitals, and physiotherapists. Most volunteers are members of the ASEM (Madrid Association of People with Neuromuscular Diseases) and FUNDAME (Spinal Muscular Atrophy Foundation Spain). Those who were interested in participating were duly informed by email or telephone along with their legal representative, if necessary. Our test design has been guided by a physiotherapist from the ASEM who was also the lead therapist of the physiotherapy section of the foundation.

The inclusion criteria were: being aged between 7 and 25 years, having a clinical diagnosis of a neuromuscular disorder, the ability to understand and follow simple guidelines and perform the required tasks, have time to use the videogame at home for about three weeks, living in the Madrid area, and the possession of a personal computer or laptop that meets the minimum requirements (64-bit, 4 GB RAM, Windows 7 or higher) and Internet access. 

The participants were divided into three groups A (*n* = 4), B (*n* = 4), and C (*n* = 6) based on muscle strength and the degree of autonomy. To assess the muscle strength, we based our procedure on the 6-stage manual muscular test proposed by Daniels, Williams, and Worthingham [31]. This test consists of measuring the force in flexion and the extension of the different limbs. Therefore, a physiotherapist holds the limb and feels the strength the patient is applying. To achieve more objective data, we preferred to use a dynamometer to measure the strength of four limbs suggested by the authors: shoulders, elbows, wrists, and torso. This measurement was done before and after the intervention. 

In addition, we evaluated the functional autonomy according to Barthel’s index that scores the ability of a patient with a neuromuscular or musculoskeletal disorder to care for himself [32]. This index is based on ten questions about basic abilities like feeding, personal use of the toilet, walking, dressing, etc. The score for each question is 0, 5, 10, or 15 points, depending on the question. The final score is the sum, so 100 BI means that the person does not need attendant care.

**Figure 5:**
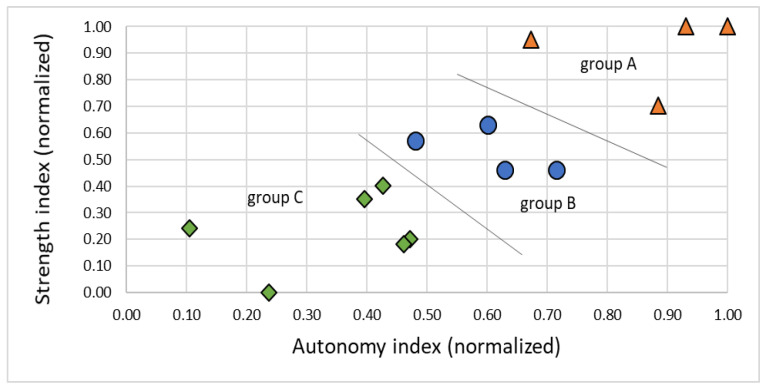
A, B, and C group classification according to strength and autonomy.

The classification of the individuals according to their strength and autonomy has been made via a clustering, as shown in Figure 5. Therefore, the values of both scales have been summed up for each subject *k* and normalized over all subjects, as shown in Equation (1):

(1) $$\bar{S_{k}}=\frac{\sum s_{ik}}{\max\left(s_{ik}\right)};\;\bar{B_{k}}=\frac{\sum b_{jk}}{\max\left(b_{jk}\right)}\;,$$

where *s_ik_* are the average values of three measurements for both shoulders, both elbows, both wrists (flexion and extension), and the torso (front and back), and where *b_ik_* are the scores obtained for autonomy (eating, bathing, dressing, etc.), each question scaling between 0 and 10. Figure 5 shows the distribution of the resulting strength index *S_k_* in relation to the Bartels autonomy index *B_k_* and the proposed classification into the groups A, B, and C.

#### 2.2.2. Intervention

The participants tried out the game at home using their personal computer and one of our Kinect cameras. On the day of the installation, a calibration program was used to capture the maximum range of the user’s movements and they tried the game with our guidance. We adjusted the difficulty parameters of each participant to the strength values measured that day. Then, they used the game without a set schedule, so we could find out how motivated they were to play without being obliged. Each player’s results and surveys were monitored daily through the web platform. In the event of a poor performance or negative feedback, the level of difficulty was reduced or we asked if a problem had occurred. The data transfer was secured using an alphanumeric identifier without a reference to personal data.

Since we did not know how long it would take each participant to finish the game, we set the trial period to three weeks. The participants were asked to reserve 2 or 3 days per week for playing, performing sessions of about 20 to 30 min on each of the days. We considered the trial to be over when either the goal of the game was reached or the player stopped playing for a more than a week and was not motivated to continue. 

Comparative trials were not planned for the following reasons:

(a) A comparison within the group would mean that the subjects played, in parallel to the big game, some comparable mini-games or did conventional exercises. This would require a fixed schedule, which contradicts our goal of letting them freely decide when and how much to play. Additionally, the testers would have needed more time and effort for the benchmark tests, which would surely have had a negative effect on the large game.
(b) A comparison with a second group (control group) would not have been possible because of the low number of available NMD patients. This is a problem in general with studies that involve NMD patients because most do not present a significant number of participants [14].

#### 2.2.3. Data Collection and Analysis

Two different types of data were gathered: (A) the scores and times achieved in the exercise scenarios, automatically transmitted to the “Blexer-med” platform; (B) three surveys: before the installation (“Pre-play survey”) for recording the demographic and personal data; after each interaction with the game (“Follow-up survey”) to get real-time feedback on participants’ performance and progress; and (C) at the end of the study (“Post-play survey”) to ask the opinion of the player and their relatives (6-point Likert scale and open questions). The three surveys were based on the proposed System Usability Scale (SUS) [33] and the Game Experience Questionnaire (GEQ) [34]. 

For the data analysis, descriptive statistics were used to calculate the demographic and clinical features. By analyzing the surveys and scores, we examined several parameters. The frequency distributions, central tendency, and standard deviations (DS) were used for descriptive purposes. When in doubt, we chose medians instead of means to avoid extreme outliers that could change the result. In addition, an analysis of variance (ANOVA) with Tukey’s post hoc test was used to compare the differences between the groups. The alpha level of a statistical significance was set to *p* < 0.05. Statistical analyses were conducted with IBM SPSS Statistics^®^ v. 26.

As some participants played or responded more often than others, it would be too imprecise to compare the players’ direct play and poll results. Therefore, we rely on the average of their responses and ratings.

## 3. Results

### 3.1. Intervention

It took six months to test the game. Starting with a small group, recruitment was carried out continuously during the test phase. Twenty-three out of forty-three eligible individuals met the inclusion criteria (age > 7 years, neuromuscular disorder, computer compatibility, and residence in the Madrid area). Nine of them showed no interest in the study, leaving 14 participants: 6 females and 8 males; aged 7–21; and average age 14.6 ± 4.5. Table 1 contains the demographic information of these people. All of them have restricted movements of the lower extremities; only two were able to play standing.

Table 2 shows the statistics about the general use of the game and compares the initial play time planned by the users with the final number of days and time actually played. The “Playing period” is the time between the first and the last day the volunteer played, not including the day of installation. The “Total number of days played” counts the number of days the game was used for.

In the “pre-play survey”, the participants optimistically reserved an average of 5.2 days a week with a mean playing time of 27.5 min (bold numbers) per session. This would have resulted in a total playing time per week of around 2.5 h. In fact, they only played 1.9 days/week, with an average playing time of 18.9 min per session. However, these times include those moments in the game when no exercise is being performed (e.g., moving Phiby inside the cell to the next obstacle, building a hut, catching an apple, etc.). The detailed analysis of the timestamps shows that for the total time, the pure training time (highlighted in bold face in Table 2) was approximately half the playing time: 43.4 min of exercising within 87.1 min of playing time. This corresponds to an average of 9.3 min of training during 18.9 min of daily playing time. 

Based on the number of days the game was available at home, we calculated an average usage rate of 36% (this was around 2.5 days per week). One participant in group A played the most; he used the game on 6 out of 8 days, which corresponds to a usage rate of 75%. This volunteer was one of three who completed the game by reaching the final goal (the exit of the valley). The other two were assigned to groups A and B.

### 3.2. Follow-Up Survey Results

The questionnaire responses were averaged for each participant to obtain a summary of the features of the whole sample. A descriptive analysis was made for the whole group. The six questions figuring in the follow-up survey are listed in Table 3 along with the results.

The survey’s ranking scale is based on a 6-point Likert scale from zero (completely disagree) to five (completely agree). Therefore, for questions Q2, Q4, Q5, and Q6, a high value means negative feedback, whereas for Q1 and Q3, a high rank indicates positive feedback. The results are generally positive since the difficulties (Q2, Q4, Q5, Q6) are rated in the lower range and “fun” and “satisfaction” (Q1, Q3) are in the upper range (between three and five).

**Figure 6:**
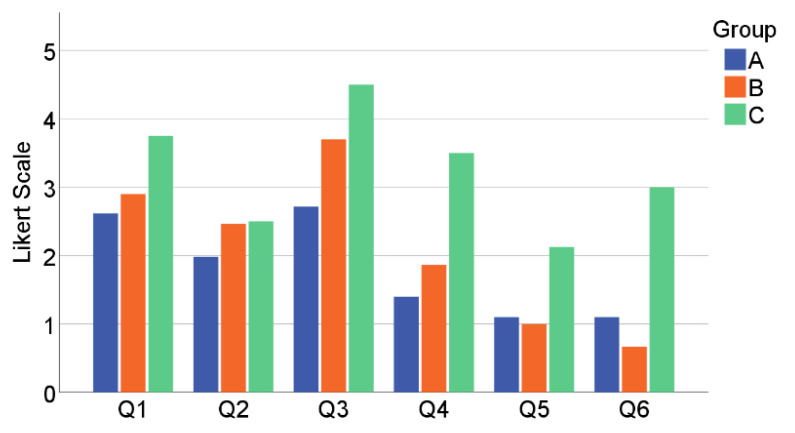
Comparison of median follow-up survey responses for groups A (*n* = 4), B (*n* = 4), C (*n* = 5, one person did not answer).

Figure 6 shows a comparison of the median values of the answers given by the three groups. It can be observed that Group C clearly stands out: they had more fun, but also tired more easily and had difficulties with the movements. It was also harder for them to achieve the goals of the exercises. One participant did not answer the questionnaire due to frustration, when the camera produced recognition problems. As we found out, this was happening because of poor lighting conditions. However, their overall satisfaction (Q3) was still the highest of the three groups.

We used the ANOVA test twice to determine if the responses differed significantly between the groups: once for the entire set of responses and once for the averages per user, because the number of surveys per user was very different. In both cases, the result was a σ > 0.05 for all questions except Q6, which means that the answers were similar for all three groups but for one exception: Q6, “goals were set too high”. Tukey’s post hoc report showed in detail that C differs from A and B, which means that two subgroups can be formed from A and B vs. C. This means that Group C had much more problems with the target values than the rest of the participants.

### 3.3. Performance Results

**Figure 7:**
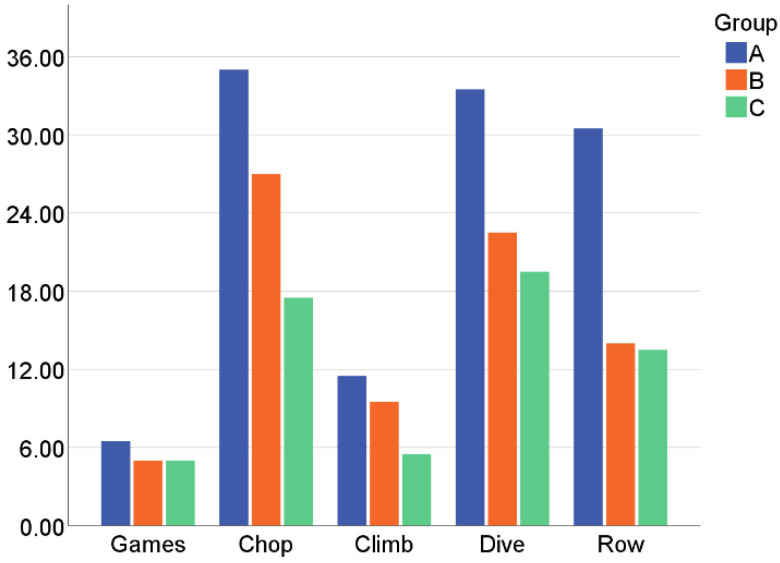
Median number of sessions and mini-games played per group.

As shown in Figure 7, the participants in group A registered a higher number of gaming sessions ($\tilde{x}=6.5$) on average than groups B ($\tilde{x}=5$) and C ($\tilde{x}=5$). When examining the individual exercises, we found that group A clearly outnumbered the others in all the exercises (${\tilde{x}}_{chop}=35$, ${\tilde{x}}_{climb}=11.5$, ${\tilde{x}}_{dive}=33.5$, and ${\tilde{x}}_{row}=30.5$) and that B (${\tilde{x}}_{chop}=27$, ${\tilde{x}}_{climb}=9.5$, ${\tilde{x}}_{dive}=22.5$, and ${\tilde{x}}_{row}=14$) did each exercise more often than group C (${\tilde{x}}_{chop}=17.5$, ${\tilde{x}}_{climb}=5.5$, ${\tilde{x}}_{dive}=19.5$, and ${\tilde{x}}_{row}=13.5$). Per session, group A completed 16 exercises on average, group B 13, and group C 10. Climbing was practiced less because it appeared less frequently in the map.

**Figure 8:**
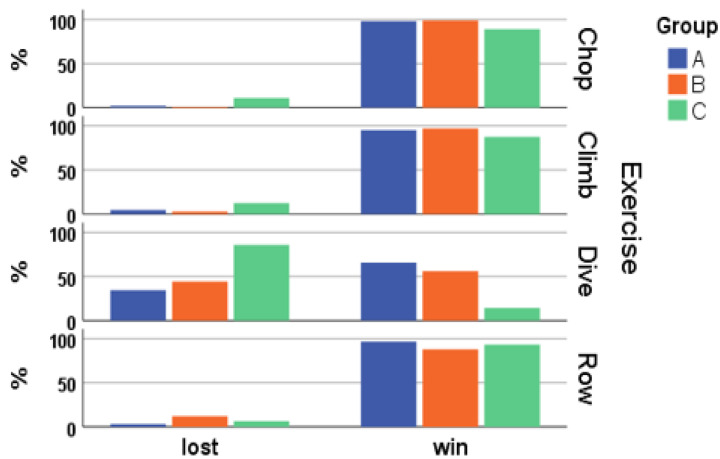
Achievements per exercise by groups. “Win” means that the score was reached in the time available, “lost” that the time had run out.

The ANOVA test was also carried out for these results, but no significant differences were found on any of the parameters studied. Figure 8 shows the relationship between the successful and unsuccessful exercises. A successful exercise means that the objective has been reached in the given time limit and we call this state “win”. If the time was too short, the exercise was not successful (“lost”). In general, the number of mini-games won exceeds the number of the lost ones, except for group C in “Dive”. Groups A and B also had difficulties, but they won more often than they lost. 

### 3.4. Post-Play Survey Results

The participants ranked the exercises by popularity from 1 to 4 (mostly liked). On average, the chopping exercise was the first choice (μ = 3.4), followed by rowing (μ = 2.6), then climbing (μ = 2.4), and diving (μ = 1.6) was by far the least popular game.

**Figure 9:**
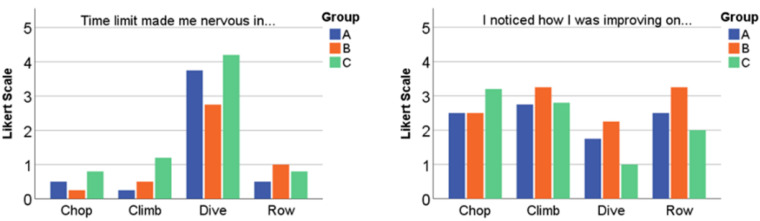
Achievements per exercise by groups. “Win” means that the score was reached in the time available, “lost” that the time had run out.

Figure 9 shows, by comparison, the perception of the positive (improvement of abilities) and negative experiences (stress) with each exercise. 

In terms of skills improvement, the results are quite similar for all groups and exercises. Different to this, the question to determine the stress factor was answered with a much higher value for the diving exercise than for the others, most notably for group C.

Finally, in Table 4, we summarize some interesting comments on the overall experience.

### 3.5. Final Assessment of Strength

**Figure 10:**
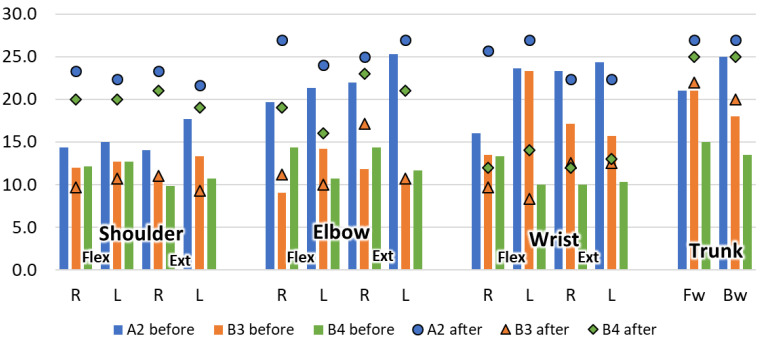
Strength measures before and after intervention.

When the participant finished their trials, we retrieved the equipment and, if they played for at least 5 days, we measured their limb strength again. In this way, we obtained the before and after measurements for three players, one from group A (subject A2) and two from group B (B3 and B4), shown in Figure 10. The playing times of these participants were: A2 = 112 min (64 min), B3 = 98 min (67 min), and B4 = 105 min (69 min); in brackets is the time corresponding to the exercises. 

## 4. Discussion

### 4.1. Performance of the Exercises “Chop”, “Climb”, “Dive”, and “Row”

The feasibility of the four virtual exercises was demonstrated in our previous study [28] and the results obtained in the present study, which involve them in a complex game, are similar: “Chop”, “Climb”, and “Row” (which correspond to the former mini-games “Whack-a-mole”, “The ladder”, and “The boat” in [28]) performed well for all user groups, with all groups showing similar success rates.

- “Chop” was played most by all the groups and produced the least stress. Group C participants (the weakest users) improved their skills most in this game.
- “Climb” was the best mastered exercise by Group B (intermediate strength).
- “Row” shows similar results as “Chop” and “Climb”, with the highest improvement rates in Group B.
- “Dive” corresponds to the “Paper bird” in [25]. Although the “Dive” exercise is somehow simpler (no arm movements are needed like in “Paper bird”), similar problems caused by the possibility to move freely around in a 3D environment, whereas in the other three scenes, the avatar maintains the same position. The movements required to control the avatar are complex and more difficult to learn, especially for group C participants who are more limited due to the wheelchair’s backrest. It has been observed that people tend to exaggerate when something does not work well, even though the game has been adjusted to very smooth movements. This type of physical control of virtual content is the biggest challenge for people with physical disabilities as it must work perfectly to avoid frustration. However, it is worthwhile to continue working on it because the 3D world expands the immersive experience enormously.

### 4.2. Motivation of the Players 

A player’s motivation and engagement in a video game can be measured using questionnaires, such as those proposed by Brockmyer et al. [35] or Ijsselstein et al. [34]. We have selected similar questions for our post-game survey and the results (Table 3) show a good general motivation above all for group C, the group that needs the most therapeutic support. 

Apart from using the questionnaires, motivation can also be measured roughly by comparing the time the users spent playing vs. the time they planned to play. As stated in the pre-play surveys, the volunteers wanted to reserve approximately half an hour on five weekdays for the game. The results show that they ended up playing less than half that time: rounded, two weekly 20 min sessions. As people generally tend to overestimate their motivation, we do not consider this result as negative. 

However, the survey shows that after some sessions, users rated Q1 (having fun) and Q3 (feeling satisfied) lower. This could be due to a certain monotony caused by the need to repeat the same exercises to advance in the game. In the post-game survey (Table 4), it was stated that the game would be funnier if it offered more types of movements and cognitive challenges. 

Style and diversity are also very important to address the interests of different users. Q1 was rated even lower if the diving exercise had to be repeated because it was not handled well, which led to frustration. The greatest satisfaction was shown when the final goal was reached (achieved by three participants). 

When comparing the groups, it is noticeable that Group A physically fully exploited the game, as the participants achieved the highest scores (Figure 8) and played most of the sessions (Figure 7), even though Group B was very close. Still, Group C also has a large benefit as the winning scores were not too much lower, the perception of their improvement was high in all exercises but diving, and, most importantly, they said they had a lot of fun and felt satisfied (Q1 and Q3). This is a very positive outcome and shows that the most affected people probably enjoyed the game most because of usually having less opportunities to play virtual games.

### 4.3. Possibilities for Physiotherapeutic Use

While a game is carried out, about half the time is spent doing physical exercises (the rest of the time the character is moved around, etc.). Thus, rehabilitation exercises could be programmed in a way such that they would be performed more frequently and over a longer time than is possible in physiotherapy sessions. This could have a positive effect on training as it reduces fatigue and increases the frequency of exercise throughout the day. 

Due to the modular architecture, the exercises could be exchanged by any others if they were more suitable for the patient.

Regarding strength, our measurements show improvements for two subjects (Figure 10) after playing for about 100 min over 5–6 days. The improvement is remarkable, but, nevertheless, two cases are not enough to show evidence. There is a need for long-term testing with a larger group of users and a rigorous testing protocol. Nevertheless, these results could indicate that the exercises embedded in the game may have a positive effect.

### 4.4. General Usability as a Personalized Exercise Program

During the tests, the “Blexer-med” platform worked as expected in terms of configurability and monitoring. The initial levels of difficulty were generally well-chosen as a starting point. In some cases, the difficulty was reduced when a player’s performance deteriorated, or he/she complained. The level of difficulty was increased when a player made very good progress in the game and performed the exercises faster and faster. Generally, a higher level of difficulty led to increased fatigue on the same day, but in subsequent sessions, fatigue was again rated lower. This may indicate that the physical condition of the individual has improved, and the player has made progress. However, this must be confirmed in future studies with suitable measurements. An interesting step would be to program an automatic and intelligent adjustment of the degree of difficulty. 

Contrary to most studies we found, our study shows results for a variety of diseases (seven different neuromuscular diseases) with a wide range of impairments (from standing to severe physical disabilities). We show that through motion amplification and the individual configuration of difficulties, similar performances and playing times can be achieved. 

### 4.5. Limitations and Future Work

Although we tried to test the prototype on a large population, it was not easy to find volunteers; only 53% of the people contacted (23 out of 43) met the inclusion criteria and, finally, only 14 participated. More studies with larger patient populations and unimpaired control groups are needed to make our results more significant and reliable.

Our study could be expanded by retesting the players after a period of use to see if their skills have improved in their daily lives. A more precise measuring system than the dynamometer and the evaluation scale would have to be used for this. However, we have not found a measuring instrument that is suitable for different diseases.

Moreover, although several previous studies mentioned in the introduction have shown the suitability of the Kinect camera for these types of applications, the device has several disadvantages: it does not provide an adequate capturing of the small movements near the body center. The presence of armrests on a wheelchair also caused the camera to confuse them with the user’s arms. Additionally, small children were not always detected well (problems in subjects C1, C2, and C3). However, some of these detection difficulties were caused by insufficient lighting and can be avoided with the Kinect v2 (Xbox One), which is more accurate, or even with the new Kinect Azure. We are currently developing a new 3D version of Phiby’s game on Unity and Kinect v2.

A total of 90% of the suggestions for improvements from the participants and their families (Table 4) relate to the “gaming experience”, the rest relate to gesture control issues. All parents confirmed that they would like to integrate an exercise game in their child’s therapy. This shows that the method is well chosen to combine personalized therapeutic exercise tools with gameplay ingredients such as immersion and flow.

## 5. Conclusions

This paper presents the first extensive tests of our exergame “Phiby’s Adventures”, which to the best of our knowledge is the first complex full-play exergame (in comparison to mini-games) configurable through a therapeutic web platform. 

The aim of this study was twofold: on one hand, we want to present an example of how to integrate different physical exercises into a large gaming environment, which involves game mechanics that engage the user and make them forget the effort of physical training. 

On the other hand, we wanted to show that the game is suitable and of use for players with very different physical conditions. Other researchers have shown that it is possible for different user groups to play and exercise together. However, as people with NMD pass through different states of limitations in their lives due to their progressive disorder, they need mechanics adapting to their respective state. This is resolved here through the adaptive adjustments of difficulty. Furthermore, NMD patients are mostly young people who are more likely to play games than the elderly, and therefore present an easily accessible group of users for VRR games. 

Our first aim has been reached: we have created a coherent map as an environment to link the existing exercises. This enables a controlled repetition of different rehabilitative movements, which can be configured for each player at a distance by the therapist. Furthermore, the game character must work on several tasks and solve problems on its way to achieve a larger goal. This is enhancing motivation and engagement. Nevertheless, a bigger variety of exercises, more cognitive challenges, and further game elements are claimed by the test persons to make the game even more interesting and appealing. 

Although our sample size is very small, with cautiousness, we could observe differences between subgroups A (best physical condition), B (moderately affected), and C (severe disabilities). Group A performed the best, but group B seemed to have greater benefits due to the adjustability and personal configurations, which are not available in commercial exergames. Group C had some problems with technical limitations due to the failing camera accuracy in poor light conditions, which led to frustration and reduced gaming time, but according to the surveys, they seem to be the ones who enjoyed playing the most.

Based on these positive findings, we recommend paying more specific attention to a game’s design when developing exergames, since this is the basis to achieve a better player involvement. We proposed an adventure game, but there is a variety of genres that also motivate gamers, such as car racing, shooters, dance moves, etc. They can captivate the attention of a user with these interests and thus motivate the individual to play and exercise more. In this way, they would especially facilitate the process of the rehabilitation of young people with different types of pathologies, since they are more eager to play.

## 6. Patents

The game and the Blexer environment led to several registrations of intellectual property for software with the following entry numbers: Blexer-med web platform 16/2019/1687; Middleware Chiro 16/2019/1576; and Phiby’s Adventures 16/2019/871.

## Figures and Tables

**Table 1:** Baseline characteristics of the participants.

Subject ID	Gender	Age (Years)	Disorder	Mobility
A1	f	15	AMC	Free walk, plays standing
A2	m	16	BMD	Free walk, plays seated
A3	f	18	Em.-Dreifuss	Free walk, plays standing
A4	m	18	BMD	Ltd. walk, plays seated
4 subjects	50% f	Ø 16.8 ± 1.5	50% BMD	100% walking
B1	m	14	AMC	Wheelchair
B2	f	17	MM	Wheelchair
B3	m	18	DMD	Ltd. walk, plays seated
B4	f	21	UCMD	Wheelchair
4 subjects	50% f	Ø 17.5 ± 2.9		25% walking
C1	f	7	SMA II	Wchr, ltd. arm lift
C2	m	7	SMA II	Wchr, ltd. arm lift
C3	m	7	SMA II	Wchr, ltd. arm lift
C4	m	14	DMD	Wchr, ltd. arm lift
C5	f	15	MM	Wchr, ltd. arm lift
C6	m	17	DMD	Wchr, no arm lift
6 subjects	33% f	Ø 11.2 ± 5.7	50% SMA	0% walking
14 subjects	43% f	Ø 14.6 ± 4.5		35% walking

AMC = Arthrogryposis Multiplex Congenita, BMD = Becker Muscular Dystrophy, DMD = Duchenne Muscular Dystrophy, MM = Mitochondrial Myopathy, SMA = Spinal Muscular Atrophy, UCMD = Ullrich Congenital Muscular Dystrophy.

**Table 2:** Playing statistics.

	Mean	Min	Max
Playing period (days) *	18.2	2.0	53.0
Total n° days played	4.4	1.0	8.0
N° days/week (planned)	1.9 (5.2)	1.0 (3.0)	5.3 (7.0)
Total playing time (min)	87.1	11.0	149.0
**Total exercise time (min)**	**43.4**	**5.0**	**78.0**
Time/day (min) (planned)	18.9 (27.5)	7.5 (15.0)	28.7 (30.0)
**Exercise-time/day (min)**	**9.3**	**4.8**	**13.7**
Rate of usage (%) **	36%	13.0%	75%

* Date of first game to date of last game played; ** days played/playing period (mean, min, and max of 14 percentage values); bold numbers highlight the pure exercising time (resting the time passing through the cells when choosing a direction).

**Table 3:** Answers of follow-up survey (averaged per participant) *.

Question	Mean	Median	Dev.	Min/Max
Q1. I had fun playing	2.8	3.0	1.4	0.5/4.3
Q2. I ended up tired	2.5	2.3	0.9	1.0/4.0
Q3. I felt satisfied	3.2	3.8	1.5	1.4/5.0
Q4. I had difficulties with movements	2.2	2.0	1.3	0.0/5.0
Q5. Time per exercise was too short	1.6	1.2	1.1	0.0/4.0
Q6. Target values were too high	1.9	1.6	1.5	0.0/5.0

* Rate of Likert scale 0–5.

**Table 4:** Suggestions for future improvements.

**Players:**
Create more types of mini-games like tennis, shooting balls, flying, paragliding, dancing…
2. Add puzzles and problems to solve.
3. Add rewards like points, medals, etc., and levels of difficulty.
4. Add different movements (legs, shoulder, head, neck…).
5. I would like to play with others, get a real score and a leader board.
6. You should improve the control, especially in the lake. Please also improve the detection of hands and arms, as these are sometimes confused with the knee or armrest of my chair.
7. The game was too childish for me.
**Parents:**
The game is an incentive to do exercises without realizing it and having fun.
2. It should be more appropriate for his/her age.
3. Control needs to be improved to include the game in therapy.
4. We would include such a game in our child’s therapy.

## Data Availability

The data presented in this study are available on request from the corresponding author. The data are not publicly available due to privacy issues.
